# An Efficient and Recyclable Catalytic System Comprising Nanopalladium(0) and a Pyridinium Salt of Iron Bis(dicarbollide) for Oxidation of Substituted Benzyl Alcohol and Lignin

**DOI:** 10.1002/open.201100014

**Published:** 2012-04-04

**Authors:** Yinghuai Zhu, Li Chuanzhao, Meriska Sudarmadji, Ng Hui Min, Algin Oh Biying, John A Maguire, Narayan S Hosmane

**Affiliations:** [a] Institute of Chemical and Engineering Sciences1 Pesek Road, Jurong Island, Singapore 627833 (Singapore) E-mail: zhu_yinghuai@ices.a-star.edu.sg; [b] Department of Chemistry, Southern Methodist University3215 Daniel Ave., Dallas, TX 75275-0314 (USA); [c] Department of Chemistry and Biochemistry, Northern Illinois University145 W. Lincoln Hwy., DeKalb, IL 60115-2862 (USA) E-mail: hosmane@niu.edu

**Keywords:** aldehydes, ionic liquids, lignin, nanoparticles, oxidations

The efficient use of renewable resources of feed stocks for the production of fuels and chemicals has become an increasingly important issue, due to the depletion of crude-oil-based raw materials.[1] This has sparked a growing interest in the development of biomass as an alternate raw material source. Lignocellulosic biomass is a renewable, abundant and sustainable resource that can be used for the large-scale production of fuels, chemicals and energy. It is made up of three main components: cellulose, hemicelluloses and lignin. The wood cells are composed of different layers, where cellulose forms a skeleton that is surrounded by other substances functioning as matrixes (hemicelluloses) and encrusting (lignin) materials. The potential use of lignin, upon oxidative depolymerization, as a renewable resource has been the focal point of our current research investigation.

Due to the insolubility of wood in common solvents, it is difficult to carry out extensive research on the utilization of wood and its components. Because of their high heat capacity, extremely low volatility, nonflammability and high thermal stability, ionic liquids (ILs) are a new generation of solvents for catalysis and synthesis and have been demonstrated to be potentially successful replacements for conventional media in chemical processes.[1] Importantly, lignin shows enhanced solubility in ILs compared to other commonly used organic solvents.[2] Thus, ionic liquids can be used as reaction media in experiments. Since there are many hydroxy groups in the lignin molecule, oxidation or oxidative cracking is possible for lignin to form aromatic aldehydes that could be useful precursors for agrochemicals or other fine chemicals.

Catalysts play critical roles in lignin conversion. The optimized catalysts should promote high conversion and suppress byproducts and char formation and condensation, while keeping the reaction severity under permissible limits. A metal based catalyst family, such as, free or supported MeReO_3_/H_2_O_2_,[3–6] porphyrin-coordinated systems, such as, porphyrin/Fe^III^/H_2_O_2_ or *m*-chloroperbenzoic acid[7–9] and porphyrin/Mn/clay/H_2_O_2_[10] demonstrate reasonable activities for the oxidation of lignin models. Interestingly, copper-and cobalt-based homogeneous catalysts are also active for this oxidation, forming the corresponding aldehydes with high selectivity.[11–13]

Transition metal nanoparticle-based catalytic systems have been found to exhibit superior catalytic activities relative to their corresponding bulk materials and often have higher selectivity when compared with conventional heterogeneous catalysts.[14] In our lab, IL-stabilized metal nanoparticles have been found to be robust and recyclable catalyst composites for organic transformations.[15] Supported nanopalladium catalysts have been used as recyclable catalysts for alcohol oxidations.[16–18] However, to our knowledge, there are no reports describing the use of IL-stabilized palladium nanoparticle-based catalysts for the oxidation of lignin models. Considering the high solubility of lignin in ILs, the use of IL-stabilized metal nanoparticles as catalysts should overcome the solubility limitation and favor the oxidative conversion of lignin to value-added chemicals, such as, aromatic aldehydes. To optimize suitable catalyst composites, benzyl alcohol was employed as a model to examine the catalytic oxidation reactions. Herein, we report our preliminary results of our investigation on a very efficient recyclable catalyst composite, comprising nanopalladium(0) and a pyridinium salt of iron bis(dicarbollide) (**5**) as a cocatalyst, for the oxidation of benzyl alcohol and lignin. Oxygen gas was used as an oxidant in our experiments.

The pyridinium salt of iron bis(dicarbollide) **5** was synthesized as described in Scheme 1. After deprotonation of 1-methyl-1,2-dicarba-*closo*-C_2_B_10_H_11_ (**1**) with *n*BuLi, the derived carboranyl anion was methylated with methyliodide to form 1,2-dimethyl-1,2-dicarba-*closo*-C_2_B_10_H_10_ (**2**) in 96 % yield. Literature methods were adopted to decapitate **2** and to form the corresponding ammonium salt [Me_3_NH][7,8-Me_2_-7,8-*nido*-C_2_B_9_H_10_] (**3**).[19] Treatment of **3** with excess sodium hydride in situ produced the dianion [7,8-dimethyl-7,8-dicarba-*nido*-C_2_B_9_H_9_]^2−^. Precipitation of the in situ-formed sodium salt Na{[π-(3)-1,2-dimethyl-1,2-C_2_B_9_H_9_]_2_Fe} with pyridinium bromide **4**15e led to the formation of iron bis(dicarbollide)pyridinium salt **5** in 78 % yield. All compounds were analyzed by ^1^H, ^13^C, and ^11^B NMR, FTIR, and MS, and the data were compared with those in the literature. All ^1^H and ^13^C NMR spectra showed peaks at expected regions. The IR spectra (Figure 1 a) of compounds **2**, **3** and **5** showed typical absorption values for *υ*_B−H_ at approximately 2500 cm^−1^. In addition, a very strong absorption band due to *υ*_N−H_ in **3** disappeared in **5**; this indicates the absence of [Me_3_NH]^+^ cation.

**Scheme 1:**
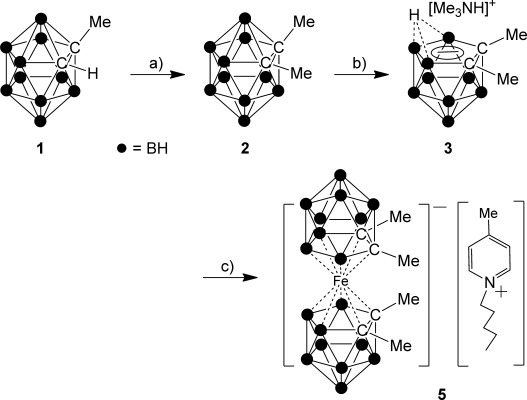
Synthesis of cocatalyst 5. *Reagents and conditions*: a) *n*BuLi (1.1 equiv), MeI (1.1 equiv), Et_2_O (30 mL); b) KOH (5 equiv), [Me_3_NH]Cl (6 equiv); c) NaH (3.9 equiv), FeCl_2_ (2.5 equiv), 1 n HCl (20 mL), 4 (2.5 equiv). For details see the Supporting Information.

**Figure 1:**
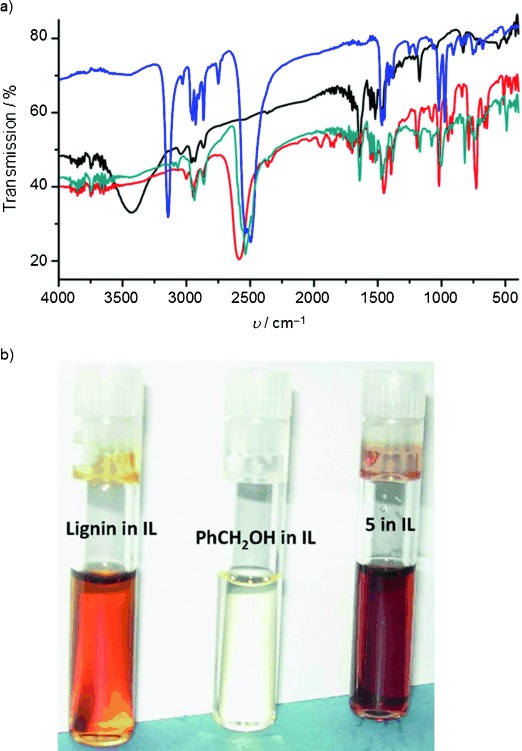
a) IR spectra of compounds 2 (—), 3 (—), 4 (—), 5 (—) and b) images of IL solutions.

Compound **5** is highly soluble in most of the commonly used organic solvents, such as, tetrahydrofuran, dichloromethane and *N*,*N*-dimethylformamide, as well as ILs, such as, 1-butyl-3-methylimidazolium hexafluorophosphate ([BMIM]PF_6_) and methylsulfate ([BMIM][MeSO_4_]). As shown in Figure 1 b, both benzyl alcohol and **5** can dissolve in a mixed IL of [BMIM][MeSO_4_] and [BMIM]PF_6_ to form a homogeneous solution.

Palladium nanoparticles as catalysts have been well investigated. The particles can be prepared conveniently by reducing various precursors. In our work, the palladium nanoparticles were prepared by reduction of H_2_PdCl_4_ with ethylene glycol in [BMIM][MeSO_4_] at 154 °C. In the procedure, after completely releasing hydrogen chloride, the pure palladium nanoparticles were obtained and characterized with transition electron microscopy (TEM) and X-ray photoelectron spectroscopy (XPS). As shown in Figure 2, the nanoparticles were very small with a narrow average size distribution of ∼2.4 nm. The XPS analysis showed typical palladium(0) absorption values at approximately 60.5 and 63.4 eV, which is consistent with the literature values.[20]

**Figure 2:**
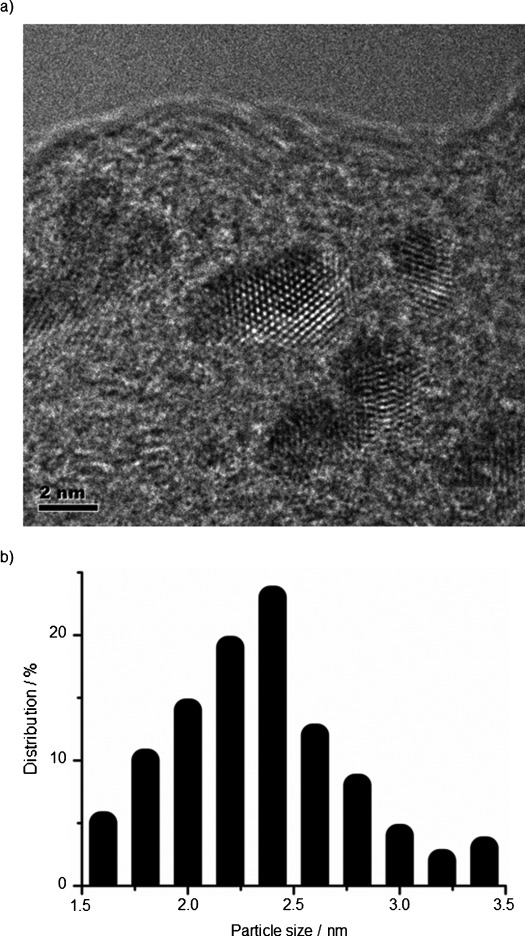
a) TEM image and b) particle histograms (120 particles counted).

The catalyzed oxidation of benzyl alcohol was performed in a mixed IL solvent of [BMIM]PF_6_/[BMIM][MeSO_4_] (2:1 v/v) at 120 °C for 18 h at 4 bar O_2_ with various cocatalysts. After reaction, the mixture was extracted with diethyl ether to obtain the crude products. The crude ether solution was concentrated and purified by thin-layer chromatography (TLC, see the Experimental Section), and the isolated pure benzyl aldehyde was identified by ^1^H and ^13^C NMR spectroscopy. The oxidation results of benzyl alcohol are summarized in Table 1. Therein, it can be seen that the IL-stabilized palladium nanoparticles are active catalysts for the oxidation of benzyl alcohol. In separate experiments, it was demonstrated that in the absence of palladium, only (2,2,6,6-tetramethylpiperidin-1-yl)oxyl (TEMPO) and **5** could generate benzyl aldehyde in yields of 13 and 22 %, respectively, according to GC analysis. Under the conditions employed in our study, the cocatalysts also play crucial roles in the oxidation reactions. After screening seven cocatalysts that are commonly used in oxidation reactions, it was found that compound **5** gave dramatically improved yields. Although the exact mechanism is not as yet clear at this point, it is possible that the high solubility of **5** in the IL media could benefit the reaction by more effectively transporting oxygen to the active metal centers. After reaction, the product mixture was extracted with diethyl ether, concentrated and purified using TLC (SiO_2_). The benzyl aldehyde was identified by ^1^H and ^13^C NMR spectra. The catalyst residue was dried in vacuo to remove any traces of benzyl alcohol and other solvents and reused for the next reactions. Under similar conditions, the catalyst composite could be reused at least five times with sustained activity (with yields of 90, 91, 90, 92 and 92 %, respectively). In addition, the concentration of palladium leached to the reaction media was found to be less than 3.4 ppm, on the basis of results from inductively coupled plasma optical emission spectroscopy (ICP-OES).

**Table 1:** Summary of benzyl alcohol oxidation.[a]

Cocatalyst	Mn 1	Co	Ce	Mn 2	TEMPO	Quinone	**5**
Yield [%][b]	41	55	47	51	76	63	93

[a] All reactions were conducted with [benzyl alcohol]_mol_/[cocatalyst]_mol_/[Pd]_mol_=100:10:1, O_2(g)_ (4 bar) in [BMIM][PF_6_]/[BMIM][MeSO_4_] (6 mL, 2:1 v/v) at 120 °C for 18 h; acetylacetone (acac), Mn 1: Mn(acac)_3_, Co: Co(acac)_3_, Ce: Ce(acac)_3_, Mn 2: Mn(acac)_2_, acac: acetylacetone, TEMPO: (2,2,6,6-tetramethylpiperidin-1-yl)oxyl.

[b] Average isolated yield of benzyl aldehyde from two reactions.

With the optimized conditions, the substituted benzyl alcohols, such as 2-methoxy, 4-methoxy, 3,4-dimethoxy and 3-phenoxy benzyl alcohols were also oxidized to the corresponding substituted benzaldehydes (see scheme in Table 2). As summarized in Table 2, the optimized catalyst composite was effective for the oxidation of substituted benzyl alcohols with reasonable yields. Since the selected alcohols contain ether bonds, which can also be found in lignin molecules, the results further encouraged us to examine the catalytic system for lignin oxidation reaction.

**Table 2:** Summary of oxidation of substituted benzyl alcohols[a]

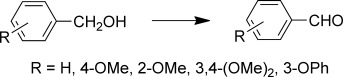	
Product	Yield [%][b]
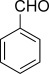	93
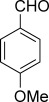	86
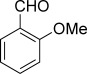	77
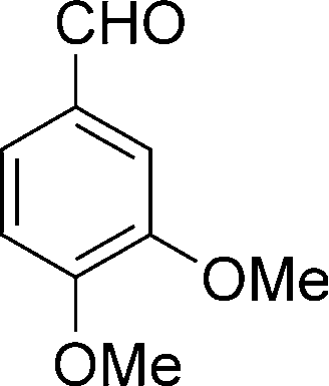	84
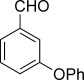	80

[a] *Reagents and conditions*: [aryl alcohol]_mol_/[**5**]_mol_/[Pd]_mol_=100:10:1, O_2(g)_ (4 bar) in [BMIM][PF_6_]/[BMIM][MeSO_4_] (6 mL, 2:1 v/v) at 120 °C for 18 h

[b] Average isolated yield of aryl aldehyde from two reactions.

Under the optimized conditions, the oxidation of lignin was examined. As shown in Figure 1 b, lignin can dissolve in IL readily to provide a homogeneous solution. Similar to benzyl alcohol, the oxidation cleavage of lignin was also conducted using nanopalladium(0) catalyst in the presence of cocatalyst **5**. Upon completion of the reaction, lignin was precipitated from the reaction mixture by adding deionized water and separated by centrifugation. It should be pointed out that in the FTIR spectra, no obvious change was found for the lignin samples before and after oxidation reaction (see Figure S-1 in the Supporting Information). The nanopalladium and water-insoluble cocatalyst **5** remained in IL [BMIM]PF_6_. Both the [BMIM]PF_6_ phase and the aqueous phase, which contained water-soluble [BMIM][MeSO_4_], were extracted with diethyl ether to collect the crude products. The aqueous phase was then combined with the [BMIM]PF_6_ phase. The resulting mixture was dried in vacuo to remove water and traces of organic solvent. The recycled catalyst was used in subsequent reactions. The ether phase was then concentrated and separated using TLC to produce four main bands (for details see the Supporting Information). The main bands were collected and structurally characterized by ^1^H, ^13^C NMR and mass spectra. The lignin conversion was calculated based on Eq. (1) as reported.[1]


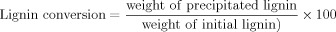
(1)

Under the conditions employed in our study, a conversion of 72 % was achieved. The major products were syringaldehyde, vanillin, *p*-hydroxybenzaldehyde, with 2,6-dimethoxy-1,4-benzoquinone as a minor product. Similar to the literature report,[1] the total mass balance is open, due to other gaseous products that could also be formed under similar conditions. The inseparable product mixtures show complicated signals in the ^1^H NMR spectra. Interestingly, the recovered catalyst composites were still active with sustained activity for at least three more reactions with a lignin conversion of 70, 65 and 68 %, respectively.

In conclusion, a pyridinium salt of iron bis(dicarbollide) (**5**) was synthesized, and its potential application in oxidation of alcohol was explored for the first time. Based on these results, it was concluded that the catalyst composite of IL-stabilized nanopalladium(0) and cocatalyst **5** was efficient for the oxidation of benzyl alcohol and lignin to produce aromatic aldehydes. Thus, this palladium(0) catalytic system was found to be efficient, robust and recyclable with high product selectivity. The broader applications of these systems and the study of the mechanism of the catalytic system are currently being explored in our laboratories.

## Experimental Section

**Oxidation of benzyl alcohol**: In a SS CAT 7 HP reactor (Hertfordshire, UK), each vial was loaded with ionic liquid (IL)-stabilized palladium nanoparticles (1 mol %), cocatalyst (10 mol %), benzyl alcohol (0.52 mL, 4.9 mmol) in a mixture of [BMIM]PF_6_ (4.0 mL) and [BMIM][MeSO_4_] (2.0 mL). The reactor was charged with O_2(g)_ (4 bar), followed by heating to 120 °C for 18 h with constant stirring. After the reaction was complete, the mixture was extracted with Et_2_O (2×15 mL). The combined ether phase was concentrated in vacuo, followed by purification with chromatography (SiO_2_, hexane/Et_2_O [1:1 v/v]), to isolate the pure benzyl aldehyde product as summarized in Table 1.

Further reaction details are available in the Supporting Information.

## Supplementary material

Detailed facts of importance to specialist readers are published as ”Supporting Information”. Such documents are peer-reviewed, but not copy-edited or typeset. They are made available as submitted by the authors.
