# Supplementary material for: An Efficient and Recyclable Catalytic System Comprising Nanopalladium(0) and a Pyridinium Salt of Iron Bis(dicarbollide) for Oxidation of Substituted Benzyl Alcohol and Lignin
Source: ChemistryOpen. 2012 Apr 4;1(2):67–70. doi: 10.1002/open.201100014 (PMC3922456; doi:10.1002/open.201100014)
Supplement: Supplementary file 1 [file open0001-0067-SD1.pdf]

ChemistryOPEN

Supporting Information

© Copyright Wiley-VCH Verlag GmbH & Co. KGaA, 69451 Weinheim, 2012

An Efficient and Recyclable Catalytic System Comprising Nanopalladium(0) and a Pyridinium Salt of Iron Bis(dicarbollide) for Oxidation of Substituted Benzyl Alcohol and Lignin

Yinghuai Zhu,*[a] Li Chuanzhao,[a] Meriska Sudarmadji,[a] Ng Hui Min,[a] Algin Oh Biying,[a] John A. Maguire,[b] and Narayan S. Hosmane*[c]

open_201100014_sm_miscellaneous_information.pdf

Experimental Section

General considerations. All operations for ligand synthesis were conducted under an argon atmosphere with glove box or standard Schlenk lines. Lignin sample (organosol, Mw = 2113, DPI = 2.68 based on GPC analysis) was purchased from Sigma-Aldrich Pte Ltd and dried at 110°C for 2 day under reduced pressure before use. Palladium atomic absorption standard solutions and other reagents were purchased from Sigma-Aldrich Pte Ltd.; trimethylammonium salt of [7,8-dimethyl-7,8-dicarba-*nido*-$C_2B_9H_{10}]^-$ (**3**) and *N*-Pentyl-4-methylpyridinium bromide (**4**) was prepared according to the literature.[1,2] ^1H, ^{13}C, and ^{11}B NMR spectra were recorded on a Bruker 400 analyzer at 400.13, 100.62 relative to $SiMe_4$ and 128.38 MHz, relative to $BF_3{\cdot}OEt_2$ respectively. Near-Infrared (IR) spectra were measured using a BIO-RAD spectrophotometer with KBr pellets technique. Inductively coupled plasma-optical emission spectroscopy (ICP-OES) analysis was determined with a VISTA-MPX, CCD Simultaneous ICP-OES analyzer. Transmission Electron Microscopy (TEM) measurements were carried out on a JEOL Tecnai-G^2, FEI analyzer at 200 kV. The MS was measured on a Thermo Finnigan MAT XP95 analyzer using EI model.

Synthesis of compound 2. At -78°C, a solution of n-BuLi (13.0 mL, 20.8 mmol, 1.6 M in hexane) was added to a solution of 1-Me-1,2-$C_2B_{10}H_{11}$ (3.0g, 18.96 mmol) dissolved in anhydrous diethyl ether (30 mL). After addition, the mixture was warmed to room temperature spontaneously and continued stirring for 4 h. MeI (1.31 mL, 20.83 mmol) was then added to the mixture at 0°C. After addition, the resulting mixture was warmed to room temperature and stirring for 1 h followed by heating to reflux for 3 h. The reaction mixture was then treated with 1*N* aqueous HCl (20 mL), and the aqueous phase was extracted with diethyl ether (2 x 50 mL). The combined ether phase was dry with anhydrous $MgSO_4$ and solvent was removed *in vacuo* to produce colorless **2** (3.15g, 96% yield). The obtained 1,2-Me_2-closo-1,2-$C_2B_{10}H_{10}$ was NMR pure and was directly used for sequent reactions without further purification. ^1H NMR ($CDCl_3$, ppm): δ 1.83-2.96 (m, br, 2 x , , 16H). ^{13}C NMR ($CDCl_3$, ppm): δ 73.32 (, 2C), 23.26 (2 x C_{Cage}-$\underline{C}H_3$, 2C). ^{11}B NMR ($CDCl_3$, ppm): δ -10.41 (d, 2B), -9.52 (d, 4B), -8.24 (d, 2B), -5.23 (d, 2B). IR (KBr pellet, cm^{-1}): ν 4078 (w, m), 3854 (m, br), 3504 (w, m), 2998 (w, s), 2943 (m, s), 2875(s, s), 2585(vs, s, $\nu_{B\text{-}H}$), 1943 (m, br), 1670 (m, m), 1450 (m, s), 1393 (m, s), 1188 (m, s), 1072 (w, s), 1020 (s, s), 937 (m, s), 728 (s, s), 644 (m, s), 527(m, s).

Synthesis of Compound 5. Sodium hydride (1.0g, 41.67 mmol) was added to a solution of of **3** (2.48g, 10.83 mmol) in anhydrous THF (40 mL). After stirring for 30 min at room temperature, the mixture was heated to reflux for about 3 h. Trimethylamine was completely removed by passing a stream of argon over the solution and through the condenser during the final 30 min of the reflux period. After cooling to room temperature, excess sodium hydride was removed by filter, the tetrahydrofuran solution of Na_2(3)-1,2-Me_2-1,2-$C_2B_9H_9$ was collected for use in subsequent reaction. To a stirred suspension of anhydrous ferrous chloride (3.5g, 27.06 mmol) in tetrahydrofurane (50 mL), was added above prepared tetrahydrofuran solution of Na_2(3)-1,2-Me_2-1,2-$C_2B_9H_9$. The resulting mixture was heated to reflux for 2 h. The mixture was filtered, the solvent was removed in vacuo, and the residue shaken with a mixture of diethyl ether (300 mL) and 1*N* aqueous hydrochloric acid (20 mL). The red ether phase was filtered and the solvent removed in vacuo. The residue was taken up in de-ionized water, from which the compound **4** (6.5g, 26.62 mmol) in de-ionized water (20 mL) was added and product **5** was obtained as deep brown solid (2.2g, 78 % yield). ^{1}H NMR (CD_2Cl_2, ppm): δ 6.72 (s, , 2H), 6.39 (s, , 2H), 4.34 (t, N--, 2H), 2.90 (s, 4-, 3H), -0.02-2.27 (m, br, N-()$_3$-, 2 x C_{Cage}-$CH_{\underline{3}}$, 2 x , -CH_2-, 39H). ^{13}C NMR (CD_2Cl_2, ppm): δ 159.85 ($\underline{C}_5H_4N$, 1C), 142.61 ($\underline{C}_5H_4N$, 2C), 128.77 ($\underline{C}_5H_4N$, 2C), 83.13 (, 2C), 61.59 (N-$\underline{C}H_2$-, 1C), 30.27, 27.37, 25.81, 21.66, 21.43, 21.25 (-($\underline{C}H_2$)$_3$-, 2 x C_{Cage}-$\underline{C}H_3$, 4-$\underline{C}H_3C_5H_4N$, 6C), 13.03 (-CH_2-$\underline{C}H_3$, 1C). ^{11}B NMR (CD_2Cl_2, ppm): δ -47.04 (br, 2B), -38.39 (d, 1B), -19.23 (d, 1B), -11.28(d, 1B), 57.58 (br, 2B), 100.63 (br, 2B). ESI-MS: m/z = 403 (M-Py+Na). M.p. 217-219°C. IR (KBr pellet, cm^{-1}): ν 3742(ms, br), 3067(m, s), 2937(s, br), 2864(m, s), 2531(vs, s, ν_{B-H}), 1639(s, s), 1540(w, s), 1457(s, s), 1373(m, s), 1170(m, s), 1069(w, s), 1004(m, s), 814(m, s), 742(w, br), 542(w, s), 486(m,s).

Synthesis of the palladium(0) nanoparticles. Nanoscale palladium was prepared by the thermolytic reduction of H_2PdCl_2 (30.0 mg, 0.120 mmol) in a mixture of [BMIM][$MeSO_4$] (5.0 mL), ethylene glycol (6.0 mL) at 154°C under argon atmosphere until the color of the mixture changed to black, and the system was cooled to 100°C, the ethylene glycol solvent was completely removed *in vacuo*, the mixture was then cooled room temperature. To isolate Pd nanoparticles, the black residue was dissolved in dichloromethane (10.0 mL) and subjected to centrifugation (5000 rpm, 40min) followed by washing with dichloromethane (2 x 10.0 mL) and drying under reduced pressure. The resulting iridium nanoparticles were subjected to analysis by XPS and TEM.

Oxidation of benzyl alcohol. In a SS CAT 7 HP reactor, in each vial was loaded with ionic liquid stabilized palladium nanoparticles in 1 mol%, co-catalyst 10 mol%, benzyl alcohol (0.52 mL, 4.9 mmol) in a mixed [BMIM]PF_6 (4.0 mL) and [BMIM] [$MeSO_4$] (2.0 mL). The reactor was then feed with oxygen gas to 4 bar followed by heating to 120°C for 18 h with continuously stirring. After reaction, the reaction mixture was extracted with ether (2 x 15 mL). The combined ether phase was concentrated *in vacuo,* followed by purification with chromatography (SiO_2, eluted with a solvent mixture of hexane/diethyl ether (v/v=1:1), to isolate the pure product benzyl aldehyde as summarized in Table 1. ^1H NMR ($CDCl_3$, ppm): δ 6.37-6.76 (m, -, 5H), 8.89 (s, -$C\underline{H}O$, 1H). ^{13}C NMR ($CDCl_3$, ppm): δ 192.25(-$\underline{C}HO$, 1C), 136.35, 134.36, 129.61, 128.92 (C_6H_5, 6C, 2C overlapped).

Oxidation of substituted benzyl alcohols. Same procedure as described above was used to oxidize other substituted benzyl alcohols. The reactor vial was loaded with ionic liquid stabilized palladium nanoparticles in 1 mol%, co-catalyst **5** (10 mol%), substituted benzyl alcohol (5.0 mmol) in a solvent mixture of [BMIM]PF_6 (4.0 mL) and [BMIM] [$MeSO_4$] (2.0 mL). The reactor was then charged with oxygen gas to 4 bar followed by heating to 120°C for 18 h with continuously stirring. After reaction, the reaction mixture was treated according to above described procedure to isolate the pure product of substituted benzyl aldehyde as summarized in **Table 2**.

4-Methoxybenzaldyhyde (585mg, 86% yield): ^1H NMR ($CDCl_3$, ppm): δ 3.95 (s, -, 3H), 7.08-7.21 (m, -, 4H), 9.97 (s, -$C\underline{H}O$, 1H); ^{13}C NMR ($CDCl_3$, ppm): δ 190.65 (-$\underline{C}HO$, 1C), 164.53, 132.12, 129.86, 114.55 (, 6C), 55.45 (-$O\underline{C}H_3$, 1C); ESI-MS, m/z = 159.04 $[M+Na]^+$.

2-Methoxybenzaldehyde (524mg, 77% yield): ^1H NMR ($CDCl_3$, ppm): δ 3.10 (s, -, 3H), 6.17-7.02 (m, -, 4H), 9.67 (s, -$C\underline{H}O$, 1H); ^{13}C NMR ($CDCl_3$, ppm): δ 189.58 (-$\underline{C}HO$, 1C), 161.77, 135.95, 128.23, 124.70, 120.53, 111.65 (, 6C), 55.52 (-$O\underline{C}H_3$, 1C); ESI-MS, m/z = 175.04 $[M+K]^+$.

3,4-Dimethoxybenzaldehyde (698mg, 84% yield): ^1H NMR ($CDCl_3$, ppm): δ 2.98 (s, -, 3H), 3.00 (s, -, 3H), 6.01-6.51 (m, , 3H), 8.89 (s, -$C\underline{H}O$, 1H); ^{13}C NMR ($CDCl_3$, ppm): δ 190.83 (-$\underline{C}HO$, 1C), 154.45, 149.59, 130.11, 126.81, 110.38, 109.08 (, 6C), 55.96, 55.14 (2 x $O\underline{C}H_3$, 2C); ESI-MS, m/z = 189.05 $[M+Na]^+$.

3-Phenoxybenzaldehyde (793mg, 80% yield): ^{1}H NMR ($CDCl_3$, ppm): δ 6.17-6.74 (m, , - 9H), 9.07 (s, -CHO, 1H); ^{13}C NMR ($CDCl_3$, ppm): δ 191.59 (-CHO, 1C), 158.39, 156.24, 138.10, 130.47, 130.08, 124.72, 124.57, 124.21, 119.50, 118.15 (, 12C); ESI-MS, m/z = 221.06 $[M+Na]^+$.

Oxidation of lignin.

Using a similar procedure as described above, lignin (200 mg) was oxidized with oxygen for 18 h at 120°C. The reaction mixture was then treated with de-ionized water (10 mL) to precipitate unreacted lignin which was then collected by centrifuge and washed with de-ionized water (2 x 5 mL) and dried *in vacuo* to a constant weight (56 mg). Based on the analysis, the lignin conversion is 72%. The water was then removed under reduced pressure, the residue was combined with insoluble phase which was then extracted with diethyl ether (3 x 40 mL), the ether phase was combined and concentrated *in vacuo,* followed by purification with chromatography (SiO_2, eluted with a solvent mixture of dichloromethane /methanol (v/v=1:1), to isolate the pure products as follows. The total mass balance is open due to the gas products may also be generated under our conditions.

Syringaldehyde (43mg): ^{1}H NMR ($CDCl_3$, ppm): δ 3.84 (s, 2 x -, 6H), 6.04 (d, -OH, 1H), 7.02 (s, , 2H), 9.69 (s, -CHO, 1H); ^{13}C NMR ($CDCl_3$, ppm): δ 190.77 (-CHO, 1C), 147.35, 140.84, 128.38, 106.70 (, 6C), 56.48 (2 x OCH_3, 2C); ESI-MS, m/z = 205.05 $[M+Na]^+$.

Vanillin (28mg): ^{1}H NMR ($CDCl_3$, ppm): δ 3.96(s, -, 3H), 6.41 (s, -, 1H), 7.05, 7.44 (d, , 2H), 9.82 (s, -CHO, 1H); ^{13}C NMR ($CDCl_3$, ppm): δ 190.99 (-CHO, 1C), 151.76, 147.19, 129.84, 127.56, 114.43, 108.82 (, 6C), 56.11 (-OCH_3, 1C); ESI-MS, m/z = 175.04 $[M+Na]^+$.

4-Hydroxybenzaldehyde (13mg): ^{1}H NMR (MeOD, ppm): δ 6.93, 7.79 (d, , 4H), 9.77 (s, -CHO, 1H); ^{13}C NMR (MeOD, ppm): δ 192.88 (-CHO, 1C), 165.19, 133.47, 130.29, 116.89, 115.85 (, 6C); ESI-MS, m/z = 145.03 $[M+Na]^+$.

2,6-Dimethoxy-1,4-benzoquinone (8mg): ^{1}H NMR ($CDCl_3$, ppm): δ 3.83(s, 2 x -, 6H), 5.86 (s, 2 x , 2H); ^{13}C NMR ($CDCl_3$, ppm): δ 186.84, 176.68 (2 x C=O, 2C), 157.31, 107.42 (2 x C=C, 4C), 56.48 (2 x -OCH_3, 2C); ESI-MS, m/z = 191.03 $[M+Na]^+$.

Other unseparable product mixtures: 11mg.

Figure S-1 IR spectra of lignin before and after reaction

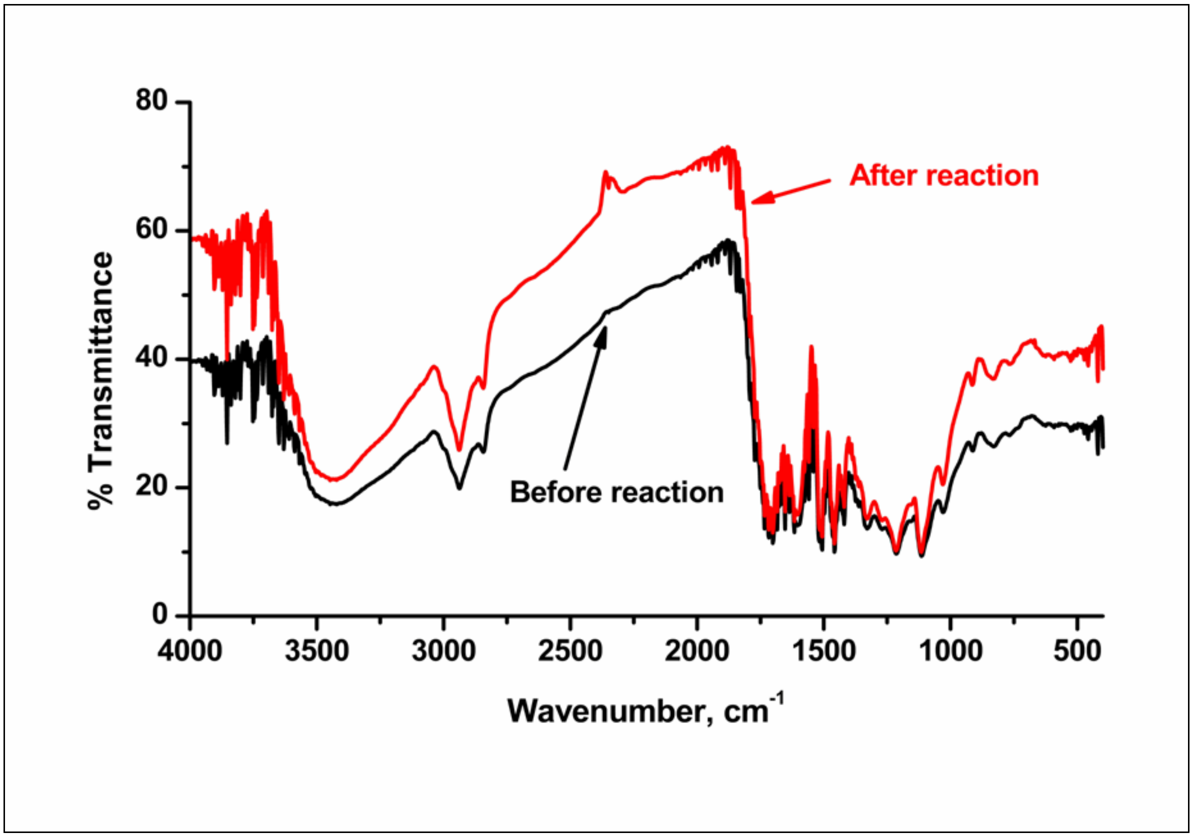

References

[1] Y. Zhu, S. H. A. Jang, Y. H. Tham, A. B. Oh, J. A. Maguire, N. S. Hosmane, *Organometallics,* **2011**, om200379c.

[2] M. F. Hawthorne, D. C. Young, T. D. Andrews, D. V. Howe, R. L. Pilling, A. D. Pitts, M. Reintjes, L. F. Warren Jr., P. A. Wegner, *J. Am. Chem. Soc.,* **1968**, *90*, 879-896.
